# Governing the Global Energy Transformation

**DOI:** 10.1007/978-3-030-39066-2_15

**Published:** 2020-03-26

**Authors:** Maria Pastukhova, Kirsten Westphal

**Affiliations:** 1 Fondazione Eni Enrico Mattei, Milan, Italy; 2 Fondazione Eni Enrico Mattei, Milan, Italy; Stiftung Wissenschaft und Politik (SWP), Ludwigkirchplatz 3-4, Berlin, 10719 Germany

## Abstract

An effective and efficient governance is key for the global energy transformation. We argue that the process under the Paris Agreement, its ‘rulebook’ and the nationally determined contributions (NDCs) will have to be accompanied by focused and tailored governance mechanisms in the energy realm. The energy sector itself is key to limiting global warming to two degrees centigrade compared to the preindustrial level, because it is responsible for over two-thirds of global greenhouse gas emissions. Yet, neither the energy transition nor energy governance start from scratch. Energy governance is already happening on many levels: the local, the national, the regional and the global. These multi-level governance structures are necessary to enable, facilitate, and accelerate the energy transition(s) on the ground. They have to be adapted, however, to the changing and transforming energy world as we argue in the conclusions. In a first step, we conceptualize the notion of ‘energy transition’ and relate it to the concept of ‘energy transformation’. We argue that it is necessary to firstly move beyond the normative and target-driven idea(s) behind ‘transition’ and to secondly bring in the systemic aspects of energy transformation. Moreover, energy security, economic efficiency, sustainability and climate neutrality have emerged over time as the guiding paradigms, forming a strategic quadrangle, as opposed to a strategic triangle, traditionally used to define energy security. In a second step, we present an overview of the current international energy governance system where multilayered governance structures have developed over time. We argue that the existing architecture is stemming from the past and is neither fit for governing the energy transition, nor even reflecting the proccesses underway in todays’ world. In a third step, we highlight that the energy *transformation* has and will have tremendous techno-economic, socio-technical and political (Cherp et al. 2018) effects that have both internal and external dimensions. Moreover, the transformation comes with (geo)political effects as it changes the political economy of energy on all levels: the global, the regional, the national and the local. In the final step, we look at ways forward. We argue that it is necessary to preserve existing multilateral institutions and to strengthen them. Moreover, we assume that governance approaches towards and inside regions will have to be re-shaped or even created from scratch. We conclude that the crumbling of the global liberal order and the crises of multilateralism are complicating the approach to a better governance of the energy transition on the global level. Moreover, we witness the emergence of illiberal tendencies in the Western democracies as well. Climate and energy are playing into the polarization of societies as the two topics emerged as a major cleavage and a conflict line. We emphasize that a just and inclusive energy transition, both on national and international levels, is necessary to keep countries and the world on a sustainable energy transformation path. The challenge faced by the planet is indeed systemic.

An effective and efficient governance is key for the global energy transformation. We argue that the process under the Paris Agreement, its ‘rulebook’ and the nationally determined contributions (NDCs) will have to be accompanied by focused and tailored governance mechanisms in the energy realm. The energy sector itself is key to limiting global warming to two degrees centigrade compared to the preindustrial level, because it is responsible for over two-thirds of global greenhouse gas emissions. Yet, neither the energy transition nor energy governance start from scratch. Energy governance is already happening on many levels: the local, the national, the regional and the global. These multi-level governance structures are necessary to enable, facilitate, and accelerate the energy transition(s) on the ground. They have to be adapted, however, to the changing and transforming energy world as we argue in the conclusions.

In a first step, we conceptualize the notion of ‘energy transition’ and relate it to the concept of ‘energy transformation’. We argue that it is necessary to firstly move beyond the normative and target-driven idea(s) behind ‘transition’ and to secondly bring in the systemic aspects of energy transformation. Moreover, energy security, economic efficiency, sustainability and climate neutrality have emerged over time as the guiding paradigms, forming a strategic quadrangle, as opposed to a strategic triangle, traditionally used to define energy security. In a second step, we present an overview of the current international energy governance system where multilayered governance structures have developed over time. We argue that the existing architecture is stemming from the past and is neither fit for governing the energy transition, nor even reflecting the proccesses underway in todays’ world. In a third step, we highlight that the energy *transformation* has and will have tremendous techno-economic, socio-technical and political (Cherp et al. 2018) effects that have both internal and external dimensions. Moreover, the transformation comes with (geo)political effects as it changes the political economy of energy on all levels: the global, the regional, the national and the local. In the final step, we look at ways forward. We argue that it is necessary to preserve existing multilateral institutions and to strengthen them. Moreover, we assume that governance approaches towards and inside regions will have to be re-shaped or even created from scratch. We conclude that the crumbling of the global liberal order and the crises of multilateralism are complicating the approach to a better governance of the energy transition on the global level. Moreover, we witness the emergence of illiberal tendencies in the Western democracies as well. Climate and energy are playing into the polarization of societies as the two topics emerged as a major cleavage and a conflict line. We emphasize that a just and inclusive energy transition, both on national and international levels, is necessary to keep countries and the world on a sustainable energy transformation path. The challenge faced by the planet is indeed systemic.

## Energy Transition—Lost in Conceptualization?

If governance of the energy transition is to be exercised effectively and efficiently, a common understanding of ‘energy transition’ seems to be helpful and necessary. Nowadays, ‘energy transition’ is a concept widely accepted and operationalized by national governments, regional and international bodies and non-governmental organizations alike. Although the term “Energiewende”^1^ has been first introduced in the early 1980s by the German Ökoinstitut (Krause 1980), it hasn’t found its way into the vocabulary of policymaking until the twenty-first century. Yet, when Germany published the “Energy Concept for environmentally sound, reliable and affordable energy supply” (BMWi and BMU 2010), which was readapted after the nuclear accident in Fukushima by the 2011 Energy Concept and the related package on the “*Energiewende*”, its English pendant ‘e*nergy transition*’ has become the international buzzword for a shift towards cleaner and more sustainable energy systems.

As omnipresent and relevant the term ‘energy transition’ might be nowadays, it is remarkably difficult to grasp, not least because of the lacking conceptual clarity and uniformity. The lack of both a comprehensive definition and a theoretical framework to support the concept of energy transition is not only lamentable from a scientific point of view: the resulting lack of common understanding among (inter)national actors also incapacitates the development of functioning international governance mechanisms to address this global issue.

**Fig. 1:**
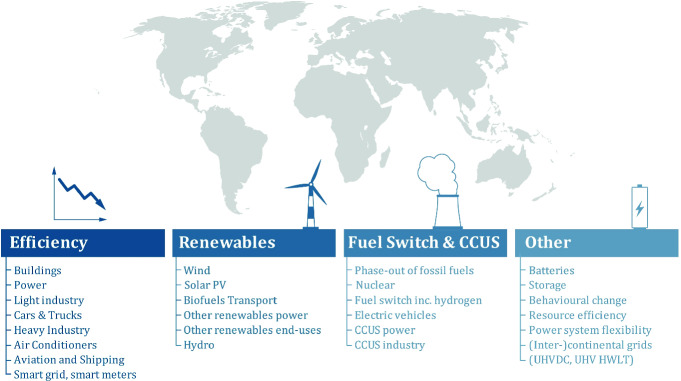
Components of energy transition(s). *Source* IEA (2019a, b) World Energy Outlook, Authors’ analysis

The main reason for this lack of conceptual integrity lies in the broadly preferred focus on the “toolbox”, that is, the single components or tasks of the energy transition. Energy transition is most commonly defined extensionally (see Fig. 1), e.g. through its components such as the increasing share of renewable energy sources in the total energy mix (IRENA, IEA), energy efficiency (EU, IPEEC, IRENA), phase-out of fossil fuels (IRENA, EU) and nuclear energy (German Fed. Gov.), electrification of the transport sector (IEA, EU), development of carbon capture and storage technologies (Norway, Saudi Arabia). An important observation at this point is, that the set of these components differs among countries, regions and organizations according to their respective agenda. In other words: the global community lacks a uniformly agreed energy transition agenda.

The respective policy approaches are guided by a set of paradigms, the central one often being *energy security*. Traditionally, in the EU and in the OECD countries, energy security has been defined through the strategic energy triangle, consisting of the three objectives of security of supply, sustainability and economic efficiency. Yet, there has always been the issue of prioritization of these objectives, given that there are not only synergies but also trade-offs between the policies addressing them. On the other side, the World Energy Council highlights that countries face an energy trilemma of addressing security of supply, ecological sustainability and energy justice simultaneously. The different wording chosen by the World Energy Council illustrates the variety of notions associated with the paradigms across the globe. While security aspects have been at the heart of energy governance since the emergence of the first energy institutions, the economic aspects have been gradually added afterwards, whereby their definition differs largely across the world. In the OECD countries, economic efficiency, competitiveness and affordability are prevailing notions. In other parts of the world, energy equity and energy justice underpin the economic, or better socio-economic angle of energy policies.

The concept of energy transition is pervasively used as a normative one, it is also often tailored to fit certain policy objectives or to underpin specific measures and steps. Therefore, international fora such as the G20 or the World Energy Council make the case for *multiple* energy transitions, i.e. structural shifts in the energy system of each country according to its respective goals and economic and resource potential (G20 2019; WEC 2014).

It is obvious that the various positions of countries in energy trading (influenced by their world market share/their position as a net importer/net exporter), in the globalized economy (trade surplus/deficit), with regard to their respective degrees of economic and social development (population growth/industrialization/urbanization) (Bradshaw 2010) as well as to the state of the energy system and the level of access to modern energy supplies determine the weighing of objectives and the prioritization of energy policy goals. With climate change mitigation, adaption and resilience added to the set of objectives, this diversity of priorities has proven to be a heavy burden and at times an obstacle for energy and climate governance beyond national levels (ibid.). Multilateral initiatives aiming to shape energy relations are in general hampered by widely scattered interests, which exacerbate the already considerable existing uncertainties. As a result, states have pursued very different pathways in energy governance: In the OECD area, it has been above all a matter of safeguarding prosperity; the post-socialist states have had to deal with the after-effects of the Soviet era, including the task of socio-economic transformation and a search for a new position in the global economy. The ‘resource curse’ and rent-seeking patterns have determined the energy dilemma of the energy-rich countries, while the question of sufficient access to energy has occupied the energy-poor countries (ibid.).

In addition to the traditional paradigms, sustainable development and growth have become key concerns. Since the second decade of the 2000s, the two key objectives of security of supply and climate protection have been accompanied by the goals of sustainable development and a fair and equal supply of energy worldwide, above all promoted by the United Nations. At that time, the United Nations also began to take an active stance on sustainable energy with its *Sustainable Energy For All* (SE4All) initiative. In the same vein, the Millennium Development Goals were translated into the Sustainable Development Goals (SDGs) in 2015. Goal 7 is to ensure access to affordable, reliable, sustainable and modern energy for all by 2030. Sustainable development is very much connected to the issues of energy justice and energy poverty, but also to environmental protection, and more specifically, protection of water, soil and air. In the same year of 2015, the Paris Agreement on Climate Change was signed. According to the Paris Agreement, countries’ ambitions on NDCs have to progress in 5-year cycles. According to Art 2.1 of the Agreement, the NDCs should be formulated in line with the goal to keep global warming well below the 2 degrees Celsius compared to pre-industrial levels and pursue efforts to limit temperature increase to 1.5°. The Paris Agreement, the consecutive Conferences of Parties (COPs) and reports by the United Nations Framework Convention on Climate Change (UN FCCC) have added a sense of urgency to the issue of climate protection, but at the same time possibly aggravated the dilemmas in addressing all four objectives even further. Local air, soil and water pollution as part of sustainable development are in many countries a major driver and mitigating climate change comes as a *transformation dividend* (Goldthau et al. 2018), rather than as a policy goal on its own. Although sustainable development has become a major underlying theme, e.g. also in the International Energy Agency and its World Energy Outlook(s), and climate is often subsumed under ‘sustainability’, we argue that both, climate and sustainability constitute paradigms in their own right.

Therefore, we suggest that a *strategic energy quadrangle* rather than a triangle is informing energy policies across the globe. Energy security, economic efficiency, sustainable development and climate change mitigation, adaptation and resilience form four major angles or baskets, to which countries associate very different notions. At the same time, however, these four angles significantly overlap and create and numerous synergies to exploit and lift.

To summarize, when it comes to energy transition governance, countries differ in terms of their starting points, their path dependencies and their future pathways as well as their ambitions. In view of this diversity, the Paris Agreement, its rulebook as well as the bottom-up mechanism of nationally determined contributions (NDCs) are important and necessary, but not sufficient preconditions to steer energy transition towards climate neutrality nor appropriate to govern energy transition(s) to meet the other objectives.

Indeed, if we are talking not about one, but multiple energy transitions, defining them through their respective components makes a lot of sense, since such a definition can be easily operationalized by, say, national policy makers. However, in order to enable global governance and international cooperation mechanisms on this issue, there must be an understanding of energy transition every stakeholder can identify itself with. Although different stakeholders propose different measures and elements, there is indeed one common element such a definition can be based on: the characteristics of the future energy system they deem necessary and aim for are the same. All major stakeholders, some explicitly (G20 2019; BMWi 2015, p. 3; G20 2019, p. 1; MOFA Japan 2018), and some implicitly (IEA 2019a, b; EC 2015; IRENA 2018; national governments, e.g. the PRC’s government^2^) define sustainability, environmental safety, economic efficiency and security of supply to be the central goals and the end-state to which the process of energy transition should lead. A future energy system with these characteristics is indeed universally aspired—the Sustainable Development Goal 7 on Energy, that is, *access to affordable, reliable, sustainable and modern energy for all* (UN 2019), has been adopted by governments of 193 countries.

Moreover, instead of energy *transition,* talking about energy *transformation* reflects the necessary systemic nature shifts in the energy system. Sometimes both concepts are used interchangeably: in IRENA’s report “Global energy transformation—roadmap to 2050” energy transformation is a means to achieve energy transition, which is conceptualized as the end-state itself: “The challenge that policy makers around the world face is how to accelerate the transition. Fully delivering the energy transition will require a transformation in how we view and manage the energy system. Transitioning in a few decades from a global fossil-fuel powered energy system, built-up over several hundred years, to one that is sustainable, will require a much greater transformation than current and planned policies (the Reference Case) envisage” (IRENA 2018: 68). As in several recent academic studies, in one if its newest reports “Geopolitics of energy transformation”, IRENA uses the term “energy transformation” intentionally instead of energy transition, to point out the broader implications a transition to low-carbon energy sources brings with it (IRENA 2019).

Against the above said, we suggest having an *intensional*^3^ *definition* of energy transition that is formulated as follows: *a policy-driven process which involves systemic shifts towards (a) sustainable and climate-friendly, economically efficient and secure energy system(s)*. The measures and building blocks of such a transition will differ from country to country. Yet, there should be a governance system behind these national efforts, to pave the way, facilitate, enhance and accelerate the energy transition(s).

## The Status Quo of Energy Governance and the Institutional Landscape

**Fig. 2:**
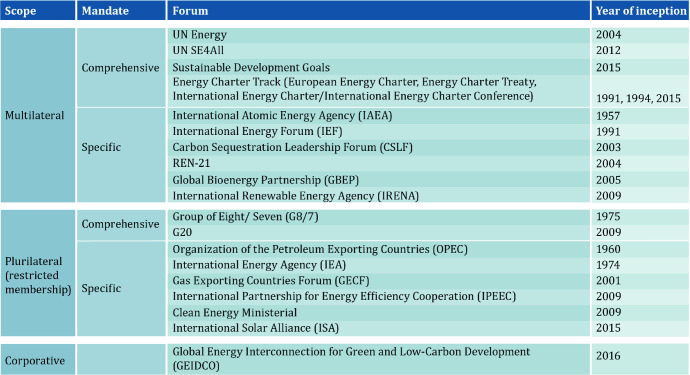
Institutional landscape: selected Governance Fora. *Source* Westphal 2015 based on Lesage et al. 2010, updated

The existing energy governance landscape began emerging in the second half of the last century and has developed over time. It is sketchy and fragmented. Within this landscape, there are very few multilateral institutions that tackle energy issues in a comprehensive way (see Fig. 2). This is the result of (1) the different positions and roles of countries in the international energy system and (2) the diverging national priorities in energy policies regarding the strategic quadrangle of energy.

The traditional organizations such as the Organization of Oil Exporting Countries (OPEC), the Gas Exporting Countries Forum (GECF) or the International Atomic Energy Agency (IAEA) focus on specific energy sources, respectively oil, natural gas and nuclear energy.

Whereas OPEC and GECF are providing platforms for dialogue and cooperation among producing countries, the International Energy Agency (IEA) was formed by the OECD countries as an organization of energy consumers and primarily in response to the first oil crisis of 1973–1974. The IEA has been dealing with different energy sources ever since, albeit it has always had a pillar on oil crisis management and prevention. The IEA has been adjusting its role constantly to the new energy and climate realities. However, its membership structure, restricted to the OECD countries, came under increasing criticism as non-OECD countries like China and India have become powerful energy market players. In face of the changing dynamics, an association process has been currently under way with major non-OECD energy powers. In 2020, the IEA comprises 30 member states, 8 association countries and 2 countries in accession. Though being a display of IEA’s adaptability, the association process is certainly an attempt to maintain the existing order.

The creation of the International Renewable Energy Agency (IRENA) in 2009 meant a significant advance, both in renewable global governance as well as with regard to multilateralism (see also Roehrkasten 2015). IRENA got a clearly defined mandate to “be the global voice and knowledge base for the use of renewable energy, to serve as a forum for international technological cooperation, and to advise the member states on these matters”. (Roehrkasten and Westphal 2013; Roehrkasten 2015). The specific focus has been on renewables. IRENA has also been looking into the geopolitical implications of an energy transformation (IRENA 2019).

As Fig. 2 shows, there are some organizations and fora that deal with specific energy sources or encompass a particular group of countries. This overview contains institutions on the global level, whereas regional organizations that specifically focus on energy (such as the Latin American Energy Organization (OLADE)) or have energy in their portfolio (United Nations Economic Commission for Europe (UNECE), European Union (EU), United Nations Economic and Social Commissions for Western Asia, for Asia and the Pacific and for Latin America and the Caribbean (UNESCWA, UNESCAP, UNECLAC), etc.) are not included here. Not included is also the World Trade Organization, which has played an important role in setting the rules for trade generally, but not in the energy sector (with an exception of energy services). The European Energy Charter in 1991 and the Treaty in 1994 were an attempt to translate similar rules into the energy trade, transport and investment. Yet, from today’s perspective, it can be said that it is very doubtful whether the Energy Charter Process can be revived and modernized in a way to provide a ‘rule book’ or a ‘code of conduct’ for international energy trade, transport and investments, despite the 2015 signed International Energy Charter.

**Fig. 3:**
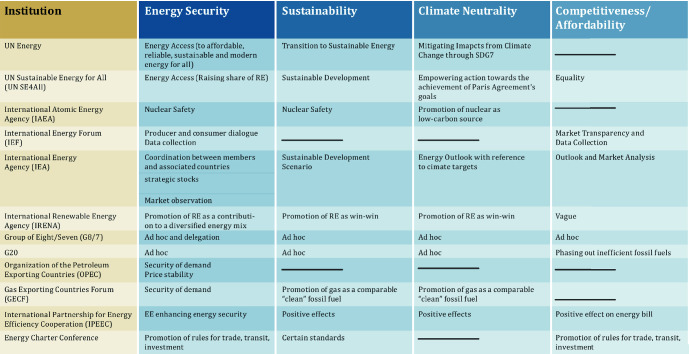
Global energy institutions, their mandate and activities. *Source* Based on Westphal 2015, updated

As Fig. 3 shows, very few existing institutions equally address energy policy objectives in an institutionalized manner.

At the end of the 2000s, there was a strong impetus to strengthen the coordination among the existing governance mechanisms and organizations. The initial idea was to better integrate the new powers such as China and India, and to have an outreach to the regions. The outreach and association process of IEA as well the International Platform of Energy Efficiency Cooperation (IPEEC) under the umbrella of IEA resulted from initiatives of the Group of Eight (G8). It was the G8 that reacted to the fact that energy governance did neither reflect the energy landscape any longer nor the changes in global politics in general. In an increasingly multipolar world, energy governance (Lesage et al. 2010) became a matter of steering committees and clubs, first and foremost of the G7/8 and G20. The G7 transformed back into an exclusive OECD-club with the crisis in and over Ukraine, when Russia was excluded from the process in 2014. The Group of 7 carried on with the agenda of tackling climate change and energy security (with the primary focus on natural gas).

In 2009, the G20 emerged as the new ‘club’ to primarily address the financial crises. The G20 began to work on energy matters under the US presidency in 2009, when G20 members declared their intent to phase out harmful and inefficient fossil fuel subsidies (Van de Graaf and Westphal 2011). This new focus was also intended as an answer to the financial and economic crises as the member countries committed themselves to a resilient, sustainable and green recovery.

The G20 is perfectly positioned to steer global energy transition. Along with the G7 countries Canada, France, Germany, Italy, the UK and the US, the G20 includes the European Union (EU), Argentina, Australia, Brazil, China, India, Indonesia, Mexico, Russia, Saudi Arabia, South Africa, South Korea, as well as Turkey. It comprises countries that are of utmost importance for a successful energy transition and includes major energy producers, consumers and key players in existing international institutions. Also, in terms of climate policies, the G20 countries would make a huge difference, if acted together, as they account for 81% of global emissions (in comparison, G7 accounts for 25%). Last but not least, the G20 includes all permanent members of the UN Security Council, and major financiers of principal international organizations.

The G20 has constantly stepped up its voluntary cooperation in energy-related areas such as subsidies, market transparency and price volatility, international energy collaboration, energy efficiency, energy access and renewable energies. The G20 summits provide countries with an opportunity to meet on an equal footing and to exchange national views and standpoints on energy topics, a major step forward being made in 2015 with the first G20 Energy Ministers Meeting that took place under the Turkish Presidency. Yet since then, every new presidency set its own priorities, which hampered continuity as an important precondition for efficient and effective energy governance. Energy ministers have met each year since 2015, except for 2017 under the German Presidency. Overall, though the G20 unites a representative group of industrialized countries and new powers that can have an impact in their respective regions, the members have very distinct and diverse policies and perspectives. This limits the role of the G20 when it comes to global energy governance and is also the reason for the group’s focus on less controversial issues, such as energy efficiency. As a result, the G20 has only partly lived up to its potential as a steering committee (Van de Graaf and Westphal 2011). In its current form, the G20 builds on the principle of voluntariness and on “soft” modes of steering, such as agenda setting, coordination among G20 members, information exchange and the steering of international organizations (ibid.). At the same time, the G20 has moved international energy governance up on its policy agenda, has rhetorically connected energy and climate policies and has enlarged its focus to sustainable development.

Since 2009, the G20 has continued to exchange on and monitor the progress towards phasing-out of fossil fuel subsidies: in 2010 the IEA, OPEC, OECD and the World Bank published reports tracking fossil fuels subsidies (IEA/OPEC/OECD/WB 2010, 2011). In 2013, the G20 endorsed a methodology for voluntary peer reviews “on inefficient fossil fuel subsidies that encourage wasteful consumption” (G20 2013, paragraph 94). Since 2013, the G20 has been addressing energy issues more comprehensively. At the 2014 G20 Summit in Brisbane, Australia, the G20 endorsed the G20 Principles on Energy Collaboration. The Chinese Presidency in 2016 continued this initiative to make energy institutions more inclusive and effective under the title “Global Energy Architecture”.

The G20 affirmed its support for the SDG target Number 7 and pledged to increase the share of renewable energy substantially by 2030. At the core of the G20 action on renewable energy is the toolkit of voluntary options, developed by IRENA. The following five options are presented as particularly beneficial for the G20 action: (1) in-depth and country-specific analyses of renewable energy costs and reduction potentials, (2) exchange good practice examples on enabling national policy frameworks, (3) development of renewable energy-specific risk mitigation instruments, (4) country-specific assessment of renewable energy technology potential and development of roadmaps and (5) support the sustainability indicators and further actions of the Global Bioenergy Partnership (GBEP), in close cooperation with IRENA and IEA Bioenergy.

In addition, G20 members decided to explore the potential for increased regional infrastructure connectivity and cross-border investment to enable greater levels of investments in renewable energy, and to continue the support for international cooperation, including capacity building for developing countries and encouraging the use of existing cooperation platforms. In 2019, energy transformation has been officially put on the agenda at the ministerial meeting on “Energy Transitions and Global Environment for Sustainable Growth” in Japan (G20 2019).

Today’s fragmented energy landscape increasingly amplifies the contours of a multipolar world. It is clear that in its current state, energy governance is far from being comprehensive, efficient and effective to steer a global energy transition. Moreover, in the current geopolitical environment the efforts to strengthen theglobal cooperation and work on global public goods seem more and more futile. At the turn of the new decade of the 2020s, personal ambitions of politicians determine politics. These are less directed to multilateral negotiations rather than to bilateral tit-for-tat zero-sum games. The volatility of personal relationships among the world leaders, where selfish nation-first policies dominate, increasingly compromises the stability and continuity of international relations.^4^

In the same vein, the global geopolitics around climate have changed fundamentally: Since the adoption of the Paris Agreement in 2015, the consensus among major powers has faded. COP 25 in Madrid in 2019 ended without a clear statement on raising the ambitions of the nationally determined contributions (NDCs), with the communiqué being watered down by the US, Brazil and Australia. The ‘NDC explorer’ shows, that the absolute majority of the countries put renewable energy first, while carbon capture, utilization and storage (CCUS) technologies are hardly mentioned. Even when taking into account that the NDCs have been produced under time pressure and that they may not be the best-grounded pledges, massive political and financial gaps are obvious and the ambitions are far too low (Pauw et al. 2019). This altogether makes energy (transition) governance—albeit granular, selective and regional—more important than ever.

## The Energy Transitions and Their Geopolitical Impact

Policymakers at all levels face the Herculean task of making energy systems more sustainable and climate friendly. Moreover, at the same time, they have to ensure the supply of, e.g. fossil fuels, for the transitional period without perpetuating the existing energy system (Westphal 2012). If one looks to the horizon 2050, in which the world aims to become carbon-neutral, the energy supply worldwide should be structured in such a way that the expected nine to ten billion people on earth have access to modern, affordable and sustainable energy supplies without further destroying the livelihoods of present and future generations (ibid.).

We assume that energy transition pathways differ and depend on countries’ respective preferences and imagined energy futures (see Chapters in this volume). Thus, we defined energy transitions as an *intensional* policy-driven process which involves systemic shifts towards (a) sustainable and climate-friendly, economically efficient and secure energy system(s).

There is no single and simple solution to transitioning the energy system(s) in line with these paradigms, as stated by the IEA in its World Energy Outlook of 2019.

**Fig. 4:**
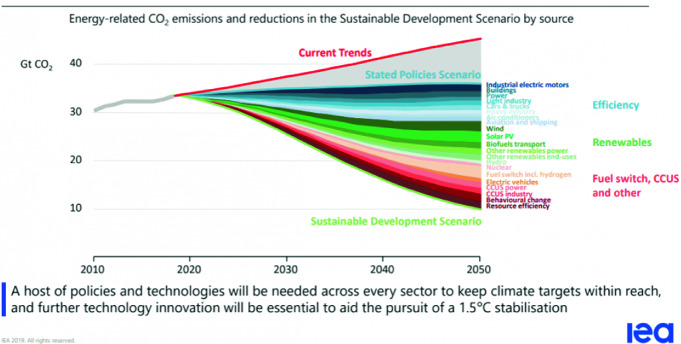
No single or simple solutions to each sustainable energy goals. *Source* IEA (2019a, b), World Energy Outlook. All rights reserved

The IEA’s WEO 2019 has been very clearly stating that there is no silver bullet at hand, but a combination of technologies ranging from energy efficiency, renewables, fuel switch, nuclear, CCUS, etc. as well as—not least—behavioural change are needed to put the world on track (see Fig. 4).

The systemic nature of energy transitions in general has been pointed out before, most notably in works on historical energy transformations (ex. Smil 2010; Kander et al. 2014, etc.). In its core, previous energy transitions have been transitions from one energy source (wood, coal, oil, electricity) and one type of energy converter (manpower, animal power, steam engine) to another. In all of these cases, major inventions of leading technologies such as the steam engine, electric lightning, etc., have kick-started processes of transition. However, all these transitions have been also accompanied by the profound and irreversible shifts on societal, ideological, political and economic levels (ibd.). In this regard, the energy transition that is taking place nowadays is no exception: it involves a shift to new, low-carbon energy sources. Yet, this time, the range and speed of the transition is and needs to be different. Moreover, the range of measures that will have to be deployed is enormous. As a result, the scale of socio-economic and political changes to be expected from the energy transitions happening around the world is not comparable to the historic cases. In other words, we have to think about energy transformation, in a way Karl Polanyi described the “Great Transformation”—neither national nor global energy systems are discrete elements. They are closely intertwined with politics, as well as with economic and social systems. A transition to a low-carbon economy doesn’t just change the energy system. It has massive knock-on and distributional effects, causing re-allocation of resources both nationally and internationally.

How to think about the New World (IRENA, 2019) and the difficult, painful, but promising transition phase? (1) The energy transition(s) come with various *structural shifts* that create new patterns of energy supply and demand, investment and data flows, new infrastructure systems and new power balances. (2) The new system will be more electrified, digitized, demand-side driven and distributed. This requires large infrastructure to adapt, to modernize or to be developed, depending on the respective countries. (3) Today’s energy system rests on individual sectors (i.e. electricity, buildings, transport, industry), each characterized by a dominant mix of (fossil) fuels (Goldthau et al. 2018). In the system of the future, the sectors (electricity, industry, heating and cooling, transport and mobility) will be coupled by the use of electricity and synthetic/decarbonized molecules and liquids. (4) As a consequence of these changes in the system, a relocation of production and demand as well as a reconfiguration of energy spaces will take place. (5) In the new energy world, the value is no longer generated primarily from the fossil fuel resource such as coal, oil or gas, but rather at the stage of conversion into end-user energy/services (ibid). In other words, more and more value will be created downstream of the energy supply chain and in services (e.g. lightning, heating, cooling, etc.). As a consequence, profits will be generated by the availability and use of low-carbon technologies.

Energy transformation does not only recalibrate energy value chains. It also (re)configures energy spaces, which are shaped by infrastructure, production chains, and industrial clusters. Energy infrastructure can be viewed as an “infrastructured” geography of “long durée” that shapes spaces and even creates its own “ecology” and topography (Högselius 2013). This is particularly true for electricity grids and their different shapes (central, decentralized) as well as sizes (local, national, trans/continental). The spatial effects of the energy transition(s) result from techno-economic change, e.g.in the shape of local micro grids or region-spanning super grids, such as those promoted by China’s Belt and Road Initiative. Connectivity will be newly defined, knocking on existing interdependencies, alleviating old sensitivities and vulnerabilities, but also creating new ones. The interconnectedness of two critical infrastructures, the electric grid and the internet creates specific challenges and hybrid threats.

If we assume that the energy transition has tremendous political, economic and social effects, the interaction with international political and geographical factor is evident (Ivleva and Tänzler 2019). Geopolitics can be understood as dynamics that stem from the interaction of geographical factors and international politics (ibid.; Scholl and Westphal 2017). In international politics energy is (intended to be) used as a tool and means to influence political outcomes, achieve foreign policy goals and as a lever to project power (Ivleva and Tänzler 2019). The geopolitics of energy transformation constitute a governance challenge in its own right. There is a growing body of literature on the energy transition having a geo-economic and geostrategic character (Bradshaw 2014; Scholten 2018; Goldthau et al. 2018; Bazilian et al. 2019; IRENA 2019).

Importantly, the very notion of energy security will change along with the transformation of energy systems: To be more precise, in the oil-centered world of the past, national security and the issue of import dependencies were at the heart of energy security. In the new energy landscape, where electrification is a major trend, the stability of the power grid will be the defining feature of energy security. Not surprisingly, energy is and will remain at the heart of national sovereignty and/or statecraft, as Daniel Yergin’s definition of energy security as “adequate, reliable supplies of energy at reasonable prices in ways that do not jeopardize major national values and objectives” hints to (Yergin 1988). Energy has always proven to be a major policy field, involving a strong role of the state, as it is closely related to its traditional tasks of providing prosperity, security and stability. The energy transition offers new opportunities to shape the energy system in a new vein, which is in line with national values and interests, while also providing and protecting global commons and goods.

While traditional geopolitics is related to power relations, the energy transition implies power shifts and alters the political economy on the national and the international levels. It creates winners and losers (IRENA 2019; Overland et al. 2019). Petrostates and coal-exporting countries are repositioning themselves in the international system as their major assets become de-valued (IRENA 2019). At the international level, fossil fuel producers are vulnerable to the fundamental changes caused by the energy transitions. Resilience to the energy transition effects depends on the percentage of fossil fuel rents in the GDP and the diversification of their economy (IRENA 2019). If petrostates such as Russia, Saudi Arabia or Iraq, etc., are confronted with declining oil rents, their socio-economic model and political systems come under severe pressure. Fossil fuel exporters are not only faced with a devaluation of their natural resources, but increasingly face fundamental challenges to their economic and social systems, since the resource wealth is part of the social contract. This in turn affects the political stability and economic growth in these countries. Yet, it is fair to say that the US shale gas and tight oil revolution has already caused a landslide by creating a new situation of energy abundance, shaking up the position of energy rich countries and diminishing their respective rents. In a sense, the US shale revolution has already anticipated certain effects for the petrostates.

Transit countries such as Ukraine, Morocco or Tunisia that gain rents from their midstream part in fossil fuel supply chains, will also feel the effects of energy transitions. Obviously, the energy transformation will have knock-on effects along the whole fossil fuel value chain making the exporting and transit countries to losers of an energy transformation. Evident winners are major importing countries, which will be able to produce more energy from renewables locally and at home or in cross-border cooperation within ‘grid communities’ (Scholten 2018), formed by political choice and not due to geological circumstances. At the same time, renewable technology leaders are emerging (IRENA 2019; Goldthau et al. 2018), gaining more and more political weight and a central place in the global markets. Hence, energy governance has to tackle the geopolitical ramifications of energy transformation and aim for a transition that is as smooth as possible. In this respect, the notion of a ‘just transition’, energy justice or evenness, is key for the global energy transition, in particular with regard to the Global South (see Goldthau in this volume).

Finally, the energy transformation has profound and even disruptive structural effects. At the national level, it inherently entails structural ruptures and puts stress on the incumbent energy system. Incumbent utilities like the German companies E. On and RWE lost significantly in their market capitalization and/or changed their asset base, which also split their renewable branches. In the socio-technical realm, a paradigm shift will have to take place, with the end consumer and/or the community moving into focus. Consumers are becoming key actors as both, consumers and producers (“prosumers”) of sustainable energy, which requires a behavioural change beyond energy saving and efficient energy use. The EU, for instance, has paid a tribute to this paradigm shift by focusing on the end consumer in its ‘Clean Energy Package for all Europeans’ of 2017. In general, this paradigm shift from supply to the end consumer has three dimensions. First, consumer behaviour is critical to the success and speed of an energy transition: consumers have to take up responsibility, be empowered and become to a certain extend ‘owners’ of the transition. Second, there is the social dimension of access, availability and affordability of (modern) energy: among others, governance measures are needed to deal with e.g. (temporary) price increases. Last, but not least, structural ruptures such as a ‘coal phase out’ are part of the decarbonization. This requires states to mitigate social risks, e.g. ones expected from the coal phase-out negotiated in Germany in 2019 (BMWi 2019). Germany’s decision to phase out coal by 2038 was achieved by a societal consensus after a long negotiation process and backed by a set of structural policies in coal regions. These policies translate into a long phase-out over almost 20 years, depending on the age (and technology used) of the respective power plants. Other countries might have the chance to leap-frog this technology, or might have to close down coal-fired power plants before their respective lifespan ends(as is likely to be the case for some countries in Asia) (IEA 2019a, b). In any case, the socio-economic dimension of the energy transformation cannot be underestimated.

As mentioned above, energy transition affects societies. Energy transition has a considerable effect on labor markets. Germany is a case in point where the narrative of creating green jobs has not really (or only temporarily) delivered. When feed-in tariffs triggered the diffusion of PV in Germany, the ‘solar valley’ in Sachsen-Anhalt boomed.^5^ Yet, as China began to expand its solar panel manufacturing industry, many involved in the same sector in Germany lost their green jobs. Socio-technical dynamics have to be closely analysed and the respective policy measures developed: In Germany, traditional energy sectors are covered by unions, primarily the respective labor union IGBCE (IG Bergbau, Chemie, Energie), whereas workers in new industries such as solar and wind energy are not organized in trade unions and therefore don’t have the same kind of support.

This particular challenge to tackle the socio-economic effects of the energy transition is thus increasingly debated as the “just energy transition”. The focus on a just transition is inextricably linked to the question of “who wins, who loses, how and why“. It is imperative to ask this question both in relation to the existing distribution of energy (e.g. “who lives with the side effects of extraction, production and generation?”), and with regards to the ongoing energy transformation (e.g. “who will bear the social costs of decarbonizing energy sources and economies?”) (Newell and Mulvaney 2013, p. 3). This, in turn, necessitates addressing the issues of distribution and access to political power, natural, social and economic resources, and the political economy behind socio-technical energy transitions (Goldthau and Sovacool 2012). Attention has to be paid to the interrelation between a just energy transition and the speed of decarbonization, though. In the beginning, mitigating social effects can be an impediment to moving ahead with the rapid decarbonization, but a sound social consensus is needed as a stabilizing element to transform the energy system. The creation of green jobs can serve as a catalyst and is even more important than social measures to compensate for income losses and job cutbacks. These social aspects are moving to the political core of many Western societies, where, i.e. resulting from such movements as “Fridays for Future”, the energy-climate cleavage has started to influence the politics and polarize the societies.

## Conclusions and Recommendations

**Fig. 5:**
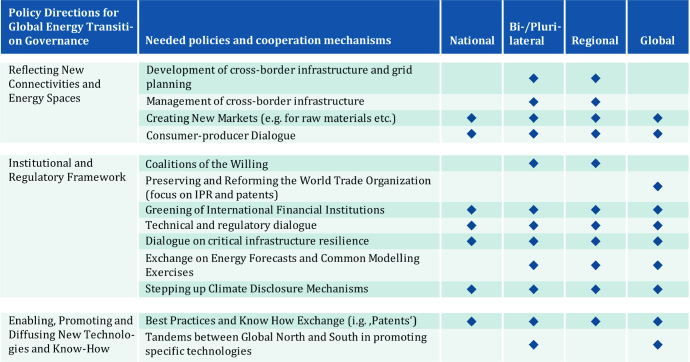
Policies and cooperation mechanisms for global energy transition governance. *Source* Authors’ analysis

The message this chapter can’t emphasize enough is that the energy transition(s) will play out differently in countries and regions, but they all will have a huge impact on all levels: the global, the regional, the national and the local. Moreover, while the targets and paradigms are in place, creating the institutional framework fit to steer the transitions’ pathways remains an open issue. As there is no simple and single solution (see Fig. 4), there is no one-size-fits-all approach to governance. Instead, what is needed is a flexible multilevel architecture which is (1) reflecting new connectivities and energy spaces, (2) enabling, promoting and diffusing new technologies and know-how and (3) adapting the institutional and regulatory framework to the changes that come along with the transformation of the energy system(s) (see Fig. 5). There is a need for a better global and regional, and a good governance on the national and local level. Particularly in today’s world of nation-first policies and geo-economics, it is imperative to establish, maintain and improve the multilateral energy landscape.

Moreover, the governance task is not only to move forward with the energy transition, and to transform the system, but also to deal with the geopolitical aspects which the energy transition brings about. Energy transformation comes with a cost, but the costs of doing nothing are higher, even if less immediate and more diffuse. The call on governance is evident, because it is assumed that this will make the transition(s) faster, smoother and more even. The transition period is assumed to have systemic transformational effects on political systems, economies and societies, and thus can be messy, disruptive and conflictive. Furthermore, the energy transition(s) are not taking place in a vacuum but have the potential to add to the geo-economic rivalry. Moreover, technology leadership and control over mineral resources can add to the struggles over political authority and power.

The global environment of great power rivalry and the crises of multilateralism are clearly complicating the global energy transformation. There is less political will to work together to create and preserve global common goods than in the past. Under these circumstances preserving the existing institutions such as WTO, IRENA and UN Energy that build on ‘altruism’ and are aiming at a level-playing field is of the utmost importance. If the global consensus is shaky, it will remain important to act plurilaterally, in clubs, coalitions and alliances. The existing governance structure (Figs. 2 and 3) can and has to ensure the functioning of today’s energy system without perpetuating it. The format of Clubs, the comprehensive institutions of IAEA and IEA will have a role to play. This is equally true for IRENA, UN Energy and SE4All, which are directed to changing the energy system(s) along the paradigms of the strategic energy quadrangle. In order to ensure that the measures undertaken (see Fig. 4) not only contribute to diversification, but in fact *transform* the system, the efforts in governing energy efficiency and renewable energy have to be stepped up. Aside from the global level, new governance structures will have to be developed or adapted at the regional level.

Without an aspiration of being comprehensive, we recommend the following focus areas.

*Governing Energy Regionalization and Connectivity.* The energy transition will reshape regions, but also create webs and routes within and between them (connectivity). So far they are largely un- or only partly governed. Yet, the existence of large infrastructure (e.g. power grids) running across regulatory spaces will require new norms, rules and standards which deal with interoperability of systems and cross-border management of flows (Scholl and Westphal 2017; Overland et al. 2016; Balmaceda and Westphal forthcoming). Energy regionalization takes place without a recognition of existing jurisdictions and polities. The question of who defines the rules of the game in the “infrastructured” space or across production and value chains is very acute. New governance schemes are necessary, also to prevent regulatory fault lines from feeding into geopolitical conflicts. This has systemic, structural and spatial implications of a transboundary nature that have repercussions on the regional and global level. Among them are the global shift of investment and financial flows due to the changes in energy and technology markets; the emergence of new geographies of demand and supply; where the digitization comes with its own risks and challenges, as both energy, IT and telecommunication sectors are connected by super infrastructures, which are highly critical to modern societies. The cascading effects in case of a crisis or a ‘black-out’ demand for specific resilience measures to be taken in smart/super grid communities. This issue is already very tangible in the EU, where electricity security and grid stability have gained utmost importance. The creation of synchronized grid communities that include Turkey, and soon the Baltics and Ukraine, come with their own governance challenges, not least of them being connected to cyber security.

*A Common Set of Global Rules.* One immediate blind spot to address in global energy governance is the lack of a code of conduct and/or a set of common rules. Both are needed in order to create a level playing field as well as transparent and functioning markets. The more technology-driven the energy system will be, the more important will rule-setting organizations such as the WTO become. Patents and intellectual property rights will remain important in order to make profits from innovation, but at the same time, solutions will be needed to provide access to important technologies for developing countries.

*Investments.* One of the key challenges for a sustainable energy transition is to get the investments right and right in time. Under the current price regime of low energy prices and in an era of abundance of energy sources, price signals to turn away from fossil fuels will be too weak or simply lacking. Investments into production sites and infrastructure predetermine and cement path dependencies given the long lead times and lock-in effects they create. Policy measures and regulatory frameworks will be key in the transition toward a sustainable global energy system. Institutions will play a central role in incentivizing and realizing the big shifts in technology, as well as creating and capturing the value and creating new business models. This is also related to the question of who will finance the necessary infrastructure. Therefore, ‘shifting the trillions’ and getting the financial and taxation framework right, is of a paramount importance. In many countries with high renewable energy and energy efficiency potential, the cost of capital is too high. New power grid infrastructure, renewable energy facilities, development of energy efficient buildings and appliances, restructuring transport sector, etc., require huge sums of infrastructure investments, great coordination efforts, and a stable regulatory framework to realize the shift. The unprecedented oil price slump triggered by the Covid-19 pandemia in the first quarter of 2020 is burning capital which will be missing in the energy sector and for the transformation as a whole.

*Technology*-*specific governance schemes and mechanisms.* Moreover, there are many issues around specific measures and technologies to be addressed by specific governance mechanisms. The table above is not considered comprehensive. Yet, it aims to visualize the complexity of the tasks faced by the global community. The instrumental and operational level aims to grasp the multitude of the technologies, components and tools needed to bring forward energy transition, among them the deployment of low-carbon energy sources, Power-to-X and synthetic fuels, but also the new approaches to power grid design (e.g. smart grids or super grids) as well as energy efficiency, sector coupling, and phase-out of fossil fuels. These elements and tools are employed altogether or in part, based on the political agenda and preferences of the respective countries—their efforts on the operational are therefore highly heterogeneous.

*A consumer*-*producer dialogue.* One of the key mechanisms to ensure a successful energy transition is an enhanced and effective consumer-producer dialogue. Such a dialogue is particularly important for depletion strategies and gradually phasing-out of fossil fuels. Moreover, this dialogue can create new partnerships to produce, trade and transport ‘de-carbonized’ molecules and fuels (hydrogen and Power-to-X) and coopt fossil fuel producers. The International Energy Forum (IEF), whose primary focus is consumer-producer dialogue, is not delivering on that. It seems that it would be more fruitful to move ahead with the ‘clubs’ and ‘coalitions of the willing’ to e.g. gradually develop hydrogen markets.

*Get the institutions right.* Steering energy transition on the global level requires enhanced technical and regulatory dialogue as well as a continuous exchange on best (and worst) practices among countries, regions and communities. There is no lack of targets, but the major challenge is to create effective incentives, frameworks and regulations to implement and accelerate the energy transformation. In addition, exchange and cooperation on a knowledge- and database on energy (including exchange on energy forecasts and common modelling exercises) must be developed, to provide transparency needed for an efficient energy transition governance. For these modes of inter- and transnational cooperation, the multilateralism of energy governance institutions should be kept up and preserved. Coordination and coherence among the existing institutions, clubs, and coalitions of the willing must be strengthened, while governance structures where cooperation on energy transition can take place in a level-playing field and is not driven by geo-economic rivalry have to be developed. For this, countries and other relevant stakeholders have to abandon the traditional perception of the quadrangle of energy security, economic efficiency, climate and sustainable development as mutually exclusive dilemmas, and put synergies from coordinating these four elements to the forefront instead.

*Tandems between Global North and South* are imperative to efficiently addressing the issues of energy poverty and just energy transition globally and to making sure that no region is left behind. With regard to the deployment of renewables, ‘tandems’ may be a way forward as pursued in the G20 with phasing-out fossil fuel subsidies. Healey and Barry (2017) rightfully highlight an “increasing inequality—of income, wealth and resource ownership” in general, and rising “inequality of access to safe and affordable energy” as well as “energy poverty” as major challenges. Energy transition regimes must address inequalities in power and injustices across entire socio-energy systems. The issue of energy justice must be incorporated into all governance mechanisms. This can be done by paying tribute to the eight principles of energy justice: availability, affordability, due process and information, responsibility, sustainability, intra-generational and inter-generational equity (Sovacool and Dworkin 2015).

*Education, research and information.* At the end of the day, the benefits of energy transition have to be communicated and pushed forward in the public debates: for instance, human security gains as improved air and water quality are among the ‘dividends’ of energy transition (Goldthau et al. 2018). Education and information are key in addressing societies in general and in particular increasingly polarized societies.

*Approach climate and energy security through the lens of public goods.* Energy transition has the potential of re-localizing the economy around human-scale enterprises rooted more closely in the communities they serve. Internally, energy transition should be ‘democratized’ as entailing a shift towards empowerment and ownership, transforming end-users into “prosumers” in the true sense of the word. Normatively, the aim should be a reconfiguration of “transition arenas” from spaces for ‘coalitions of frontrunners’ towards more open spaces for deliberation, dialogue and participation (Barry et al. 2015). For this, approaching energy and climate security through the lens of global public goods as opposed to a strategic national interest is important.

We conclude that a just energy transition on all levels, but certainly on the national and the international level, is necessary to keep countries and the world on a sustainable energy transition path. It is essential for the international community to stick to the Paris Agreement in order to keep up the level of ambition nationally. But also vice versa, the international ambition cannot be sustained without an enduring social consensus in major countries.

## Footnotes

1 German for “Energy Transition”.

2 As stated, for instance, in Xi Jinping’s speech at the 19th National Congress of the Communist Party of China (Xi 2017).

3 An intensional definition provides the meaning of an expression by specifying necessary and sufficient conditions for correct application of the expression. An intensional definition should be distinguished from an extensional definition, which merely provides a list of those instances in which the expression being defined is applicable. Cook, Roy T. "Intensional Definition". In *A Dictionary of Philosophical Logic*. Edinburgh: Edinburgh University Press, 2009. 155.

4 We owe these thoughts to Carlos Pascual and his inspiring presentation in December 2019 in Berlin.

5 https://www.mdr.de/sachsen-anhalt/dessau/bitterfeld/solar-valley-solarzellen-photovoltaik-chronologie-q-cells-solibro-100.html.
